# Sentinel lymph node identification by blue dye in patients with breast carcinoma

**DOI:** 10.12669/pjms.322.9563

**Published:** 2016

**Authors:** Nighat Bakhtiar, Farhat Jaleel, Foad Ali Moosa, Naeem Akhtar Qureshi, Masood Jawaid

**Affiliations:** 1 Dr. Nighat Bakhtiar, MBBS. Post Graduate General Surgery Fellowship Trainee, Department of Surgery, Dow International Medical College/ Dow University Hospital, Dow University of Health Sciences, Karachi, Pakistan; 2 Dr. Farhat Jaleel, MBBS, FCPS (General Surgery). Associate Professor, Department of Surgery, Dow International Medical College/ Dow University Hospital, Dow University of Health Sciences, Karachi, Pakistan; 3 Prof. Foad Ali Moosa, MBBS, FRCS. Professor & Head of Department of Surgery, Dow International Medical College/ Dow University Hospital, Dow University of Health Sciences, Karachi, Pakistan; 4 Dr. Naeem Akhtar Qureshi, MBBS, FCPS (General Surgery). Assistant Professor, Department of Surgery, Dow International Medical College/ Dow University Hospital, Dow University of Health Sciences, Karachi, Pakistan; 5 Dr. Masood Jawaid, MBBS, MCPS, MRCS, FCPS (General Surgery), MHPE Visiting Faculty, University of Health Sciences, Lahore, Pakistan

**Keywords:** Breast cancer, Diagnostic accuracy, Methylene blue, Sentinel lymph nod

## Abstract

**Objective:**

To determine the diagnostic accuracy of methylene blue dye to detect axillary lymph node metastases in patients with breast carcinoma by taking histopathology as gold standard.

**Methods:**

This quasi experimental study was done at Department of Surgery of Dow University Hospital Karachi during January 2013 to September 2015 after the approval of Hospital Ethical Committee. A total number of 85 patients with biopsy proven carcinoma were included in the study.1% methylene blue dye was infiltrated in the peri tumoural area of the diseased breast. The blue stained node called sentinel lymph node (SLN) was recognized and carefully dissected out. SLN and mastectomy with axillary clearance specimen was sent for histopathology in two separate bottles and the report of the histopathology was compared.

**Results:**

The axillary lymph nodes were positive for carcinoma in 61 cases out of 85(71.7%). Two of the patients had negative sentinel lymph node but positive non sentinel lymph node (false negative), and in three cases sentinel lymph node were involved only but not the rest of the axilla (False positive). The sensitivity, specificity and accuracy were 96.8%,86.36% and 94.1% respectively.

**Conclusion:**

Methylene blue dye technique is a reliable and safe diagnostic modality for detection of Sentinel lymph node in breast cancer patient because of its high accuracy.

## INTRODUCTION

Breast carcinoma is the most common cancer of women worldwide including 23% of all female cancers.^1^ In Pakistan it isaccounted for 23% of all and 41% of female cancers.^2^ Histopathology is considered as the Gold standard for the diagnosis of Breast cancer.^3^ The axillary lymph node status is a significant measurement to plan the consequent adjuvant treatment of breast carcinoma and is also the most important prognostic factor. It is assessed by sentinel lymph node (SLN) biopsy which is defined as the lymph node that receives lymphatic drainage from tumorearliest^4^ therefore this node has the highest chances to containmetastatic breast cancer.

Sentinel lymph node biopsy is a least invasive surgical method to stage axilla and it also decrease the morbidity of axillary clearance.^5^ SLN biopsy by radio colloid method was first reported in 1993 by Krag et al.^6^, and by blue dye method by Giulianoet al.^7^ in 1994. Combined use of radioactive colloid and blue dye injection is considered as gold standard for axillary sentinel lymph node biopsy (SLNB) in breast cancer with 97% accuracy rate,^8^-^10^ but this combine usage does not attain an adequately higher detection rate to defend the cost.^11^ While some researchers have been using blue dye only for identification of SLN with good reliability.^12^ The positive results found by using methylene blue dye and by isosulfan blue dye were 99% and 97% respectively.^13^, ^14^ While another similar study for methylene blue dye was done showing the sensitivity and specificity of 85.7% and 71.4% respectively.^15^ So the efficiency of detecting SLN by Methylene blue is as good as Isosulfan blue with cost effectiveness and is equal to ALND in breast cancer, but there is difference between the percentages of positive results in different studies.^15^

The aim of this study was to identify the sentinel lymph node correctly by methylene blue dye which will permit in future to leave out the preventable axillary dissection in clinically node negative patients thereby decreasing morbidity and expenditure of this procedure. This study with large sample size as compared to the previous studies done in Pakistan^16^ will help in the improvement of optimum management of node negative breast cancer patients at Dow University Hospital.

## METHODS

This quasi experimental study was done at Department of Surgery of Dow University Hospital Karachi during January 2013 to September 2015 after the approval of Hospital Ethical Committee. A total number of 85 patients were included in the study. This sample size has been calculated using the formula^17^, based on sensitivity at 95% confidence level and acceptable margin of error set at 10%. All patients with true cut biopsy proven carcinoma breast, coming through outpatient departments and all those who were willing were planned after taking informed and written consent for modified radical mastectomy as their surgical treatment were included. Patients with metastatic, inoperable disease, previous breast surgery, clinically palpable nodes, larger tumor size or those who were not willing for the procedure were excluded from the study. All surgeries were done by the same senior general surgeon of the hospital. Non probability consecutive sampling was done.

At the operation table, prophylactic antibiotics were given at the time of induction to all the patients and after draping 3-5ml sterilized 1% methylene blue dye was infiltrated with a 10cc syringe in the peri tumoural area of the diseased breast. Gentle massage of breast was done for 1-2 minutes and then after 10 minutes dissection was done in axilla for localization of sentinel lymph node by giving incision in the axilla that incorporates the incision of Modified Radical Mastectomy. The blue colored lymphatic channels were followed which lead to the blue stained node called sentinel lymph node (SLN), the node was recognized and carefully dissected out, and any other stained node if found in vicinity was also removed.

Following that, in all patients the routine modified radical mastectomy with axillary clearance was performed and the SLN and mastectomy with axillary clearance specimen was sent to the histology laboratory fixed in formalin in two separate bottles marked “A” and “B” respectively and the report of the histopathology was compared.

Data was analyzed by utilizing SPSS version 17. Mean and standard deviation were calculated for age tumor size and weight. Frequency and percentage were calculated for gender, breast involvement and quadrant of breast involved, accuracy of Methylene blue dye. A 2×2 table was constructed to calculate sensitivity, specificity, PPV, NPV and diagnostic accuracy of Methylene blue dye to predict axillary status taking Histopathology as gold standard. Effect modifier was controlled through stratification of age, gender, weight, size of tumor, breast involvement and quadrant of breast involvement to see the effect of these on outcome variables.

## RESULTS

Total 85 patients were included in the study of age ranging between 23-70 years. The mean± SD age was 45.7 ± 1.0 years. The age group mostly involved was between 45-56 Years. The tumor was present in superiolateral quadrant in 76 patients (89.4%), in superiomedial quadrant in four patients, three had tumour in inferiomedial and two had in inferiolateral quadrant.62% of patients had carcinoma on right side while 38% of cases had it on left side.31.8% (n=27)presented with T1 tumor(<2cm in size) while 45.9% (n-=39) of patients had T2 tumor (2-5cm in size) 22.4% (n=19) patients had T3 (>5cm) tumour shown in Table-I. Histopathology of all patients came out to be infiltrating ductal carcinoma except one case which was lobular carcinoma.

**Table-I:** Size of the tumor (n=85).

Size of tumor in cm	No. of Patients (%)
<2 cm (T1)	27 (31.8%)
2-5 cm(T2)	39 (45.9%)
>5cm(T3)	19 (22.4%)

The axillary lymph nodes were positive for carcinoma in 61 cases out of 85(71.7%). Two of the patients had negative sentinel lymph node but positive non sentinel lymph node (false negative), and in three cases sentinel lymph node were involved only but not the rest of the axilla (False positive). The Values of the test and the disease positive with its sensitivity, specificity and accuracy of the technique are shown in Table-II.

**Table-II:** Values of the test and the disease positive (n=85).

	Histopathology +	Histopathology -	
SLN Positive	61	3	80
SLN Negative	2	19	21
	63	22	85

Sensitivity: 61/63 × 100 = 96.8%

Specificity: 19/22 × 100 = 86.36%

PPV: 61/80 × 100 = 76.25%

NPV: 19/21 × 100 = 90.4%

Accuracy: 85/80 × 100= 94.1%

## DISCUSSION

The most favorable method for SLN identification and its biopsy has always been under considerable discussion. The experience of surgeon is always increased in the accurate identification of Sentinel lymph node. The sensitivity, specificity and accuracy in this study were 96.8%, 86.36% and 94.1% which are comparable to other studies which showed 83- 100%, 100% and 92-100% respectively.^17^-^19^ Same as the positive and negative predictive values of other studies were 75% and 83.33% respectively in comparison with our study which showed 76.25% and 90.4%.^20^

The total number of patients in this study was 85 which is a good number as compared to the other studies done in same country i.e Vohra et al.^14^ included 30 patients in their study. Rate of negative SLN and axilla was reported 62% by Kebudi et al.^21^, 70% by Zaman etal.^22^ and 53.3% by Vohra et al.^14^ while in this study 20 (74.07%) patients with T1 tumour had negative SLN and axilla for metastasis.

Possible explanation for this variation was explained by Guilliano et al. in 1994 was the widespread penetration of the tumor which lead to re-distribution of the lymph fluid to the non-sentinel nodes or may be the early practice of the surgeon. In his study which included 174 patients and it illustrated (65%) identification and (12%) false negative rates, but when the same study was repeated with the same group in 1997 showed (93%) identification and (0%) false negative rates proved that it the reason behind was the learning curve for the procedure.^7^ This point of view was also explained by other researchers who explained that this method has a distinct but a short achievable learning curve and this will eventually accomplish better identification and improved false negative results.^23^

Pakistan being developing country has a very a profound financial burden on its health management system because of its increasing population including more than 50% females who have high risk of carcinoma breast. Therefore it is now very important to do research regarding this serious issue in cost effective way. This study has showed the advantage of using blue dye in a non affording population having less equipped hospitals by its good accuracy rates without using complicated and expensive devices like Gamma camera and frozen sections which are not available in most of the hospitals.^24^ Besides that perseverance on frozen section in less equipped hospitals will also face the problem of transfer of SLNB to a well equipped hospital.

## CONCLUSION

Methylene blue dye technique is a reliable and safe diagnostic modality for detection of Sentinel lymph node in breast cancer patient because of its high accuracy. It is a precise, readily available and cost-effective method to assess the metastatic status of axillary lymph nodes. Morbidity of patients will be decreased in future when sparring of axillary lymph nodes will be done after achieving the short learning curve of surgeons.
